# Investigating the impact of fuel price shocks on bicycle sharing usage in Budapest

**DOI:** 10.1038/s41598-024-68677-0

**Published:** 2024-08-07

**Authors:** Zombor Berezvai, Vincenzo Basile, András Kálecz-Simon, Barna Bakó

**Affiliations:** 1 Corvinus University of Budapest, Budapest, Hungary; 2 Federico II University of Naples, Naples, Italy

**Keywords:** Bicycle sharing systems, Fuel price increase, Hungary, Sustainability, Climate-change mitigation

## Abstract

The creation of sustainable urban communities is contingent upon the establishment of a sustainable, efficient, and fast transportation system. Bicycle sharing systems (BSS) are one of the most sustainable and inclusive ways of transportation in cities. An important question is how to increase BSS ridership and whether it can effectively replace cars in cities, hence contributing to achieving the 11th Sustainable Development Goal and creating sustainable urban communities. This paper aims to contribute to this stream of research by investigating the effect of fuel prices on BSS ridership. We exploit a natural experiment from Budapest, Hungary, where fuel prices were capped between November 15, 2021, and December 6, 2022. Once the price cap was suddenly eliminated, fuel prices increased by around one-third immediately leading to a very substantial and rarely observable one-time price increase. The difference-in-difference regression results indicate a 2–6% increase in BSS ridership after the elimination of the fuel price cap. The geographical pattern of the change shows that BSS usage mainly increased in the outer part of the city; however, some areas observed a decline. The regression results are also reinforced by survey findings. Understanding these dynamics is crucial for effective urban planning and transportation policymaking.

## Introduction and literature review

Sustainable transportation holds a vital position within the 11th Sustainable Development Goal (SDG), as the resilience and viability of cities and human settlements hinge on the presence of well-functioning transportation systems and supportive commuting habits^1–3^. The creation of sustainable urban communities is contingent upon the establishment of a sustainable, efficient, and fast transportation system. It is evident that bicycles and bicycle sharing systems (BSS) have a significant part to play in this transformation.

The current dominance of motorized transport in urban settings gives rise to a host of challenges, including air pollution, traffic congestion, inadequate parking infrastructure, substantial CO_2_ emissions, and noise pollution. These also lead to substantial negative impacts on the environment^4^. These challenges not only degrade the quality of life for urban residents but also pose long-term threats to public health and the sustainability of urban ecosystems and communities. While technological advancements in transportation offer promising solutions to mitigate some of these issues, they often require significant infrastructural investment and may not address all predicaments associated with motorized transport.

In response to the shortcomings of traditional motorized transport, micromobility solutions such as bicycles and scooters have gained traction as viable alternatives for short-distance urban travel^5^. These modes of transportation offer numerous advantages over traditional vehicles, including lower emissions, reduced noise pollution, and increased space efficiency. By promoting the adoption of micromobility solutions, cities can reduce their reliance on fossil fuels, mitigate air pollution, and improve overall urban liveability^6^. Policy initiatives aimed at promoting cycling and other forms of micromobility have gained momentum in recent years, reflecting a growing recognition of the importance of sustainable transportation in urban planning and development^7^.

Shared micromobility services, including BSS have emerged as key components of sustainable urban transportation networks, propelled by advancements in technology and shifting urban mobility trends. Modern bicycle sharing platforms integrate sophisticated features like GPS tracking, mobile apps for booking and payment, and even smart locks for enhanced security. These systems operate under various business models, including government-funded initiatives, public–private partnerships, and fully private enterprises. These systems have been widely adopted in cities worldwide, with research indicating their effectiveness in reducing car dependency, alleviating traffic congestion, and improving overall transportation efficiency^8^. By integrating BSS with existing public transit options, cities can further enhance the accessibility and reach of their transportation networks, addressing the “first-and-last-mile” problem and encouraging modal shifts towards more sustainable modes of travel^9^. The improvement in connectivity can further have economic benefits^6^.

Although BSS has many positive aspects regarding urban sustainability, a constraint needs to be mentioned here. Namely, BSS typically replaces trips that would otherwise be made by public transport, potentially leading to conflicting relationships with non-motorized modes of transportation. Studies by Martin and Shaheen^10^ or Munkácsy and Monzón^11^ shed light on these complex dynamics, highlighting the need for a nuanced approach to integrating BSS into urban transportation networks.

Several factors influence the usage and adoption of BSS. Weather conditions, convenience, safety perception, and the availability of dedicated cycling infrastructure are among the key determinants of BSS usage rates^12–14^. The Covid19 pandemic also had significant effects on bicycle usage frequency, pattern, and motivation^15–17^.

Additionally, fuel prices play a significant role in shaping travel behaviour, with studies indicating that higher fuel prices can lead to increased bicycle usage as individuals seek cost-effective alternatives to car travel^18,19^. However, the relationship between fuel prices and bicycle usage is complex and multifaceted and influenced by various factors. As mentioned above, weather conditions, convenience, safety perception, urban infrastructure, and the availability of dedicated cycling lanes may all interact with fuel prices to shape the decision-making process of potential bicycle users. Pucher and Buehler^19^ conducted a comparative study of BSS usage in the US and Canada, identifying several factors that influence shared bicycle trips. Alongside weather conditions and cycling safety, they found a significant relationship between trip volume and gasoline prices. Higher fuel prices were found to lead to an increase in shared bike trips, as individuals sought cost-effective alternatives to car travel. Furthermore, the study conducted by Frondel and Vance^13^ found that an increase in fuel prices led to a higher probability of bicycle use, with this relationship being notably stronger in urban areas. Similar results were presented in He et al.^18^. Their results suggest that a surge in fuel prices significantly influences not only trip frequency but also trip duration, with a noticeable increase in short trips. These findings highlight the role of economic incentives and the potential for BSS to thrive in response to rising fuel costs.

Comparisons between different means of micromobility have also shed light on the influence of fuel prices on shared bike systems. Younes et al.^20^ used data from Washington, DC-based scooters and BSS providers to compare various factors influencing the number and duration of trips. Their findings indicated that BSS users were more sensitive to weather conditions than scooter users. Although all users were affected by fuel prices, shared bike usage volume demonstrated a lower degree of responsiveness to these changes compared to scooters. This suggests that BSS may be perceived as a more stable and reliable mode of transportation, with users exhibiting less fluctuation in response to fuel price variations.

Additionally, studies have explored the impact of fuel shocks, such as fuel shortages, on bicycle usage, shedding light on how short-term disruptions can influence long-term behavioural patterns. For example, Barriola^21^ examined the effects of fuel shortages on shared bicycle usage in Mexico City and Guadalajara. By investigating the impact of supply chain disruptions on BSS trips, the study provided insights into the dynamics of usage patterns under challenging conditions. During the fuel shortages, there was a significant increase in BSS trips in both cities. However, in the longer run, trip volumes returned to normal in Mexico City, whereas Guadalajara experienced continued growth in both the number of users and trips even after the fuel shortage was resolved. Although the study did not provide a definitive explanation for this difference, it highlighted two distinguishing factors between the cities. Mexico City had numerous alternative transportation options available, which may have diminished the long-term effects of the fuel shortage on the BSS. Additionally, usage patterns in Guadalajara exhibited a more balanced distribution across docking stations, indicating the importance of designing a well-balanced BSS to foster usage growth.

In this article, we focus on investigating the impact of a unique fuel price surge in Budapest, Hungary, on the utilization of the city’s BSS. Following the abandonment of a fuel price cap regulation, fuel prices in Hungary experienced a notable and unprecedented one-time increase of approximately one-third of the price. This sudden price surge provides a rare opportunity to examine the immediate and short-term effects (up to 7 months) of fuel price changes on BSS ridership patterns.

By leveraging data from the Budapest BSS, we aim to assess the immediate changes in ridership following the fuel price increase and evaluate its sustained impact over time. Additionally, we seek to analyse the geographical distribution of BSS usage across different areas of Budapest to identify specific areas where the effects of the fuel price increase were particularly pronounced.

The paper is structured as follows: Section "The case of Budapest" provides a short overview of Budapest and the Hungarian fuel price cap regulation, followed by the introduction of data and methodology in Sections "Data" and "Methodology", respectively. Section "Results" presents the results, Section "Discussion" discusses the findings with policy implications, and Section "Conclusions, limitations, and future research" concludes by summarizing key insights and suggesting avenues for future research.

## The case of Budapest

Budapest is the capital city of Hungary with a population of approximately 1.7 million. However, taking also the suburb region into consideration, the population is above 2 million. From a transportation point of view, the modal split is dominated by sustainable transportation modes with a public transport share of 47%, a walking share of 16% and a bicycle share of 2%. The remaining 35% of the trips are made by private cars as reported by the Budapest Mobility Report 2021 https://bkk.hu/downloads/24709/).

**Figure 1:**
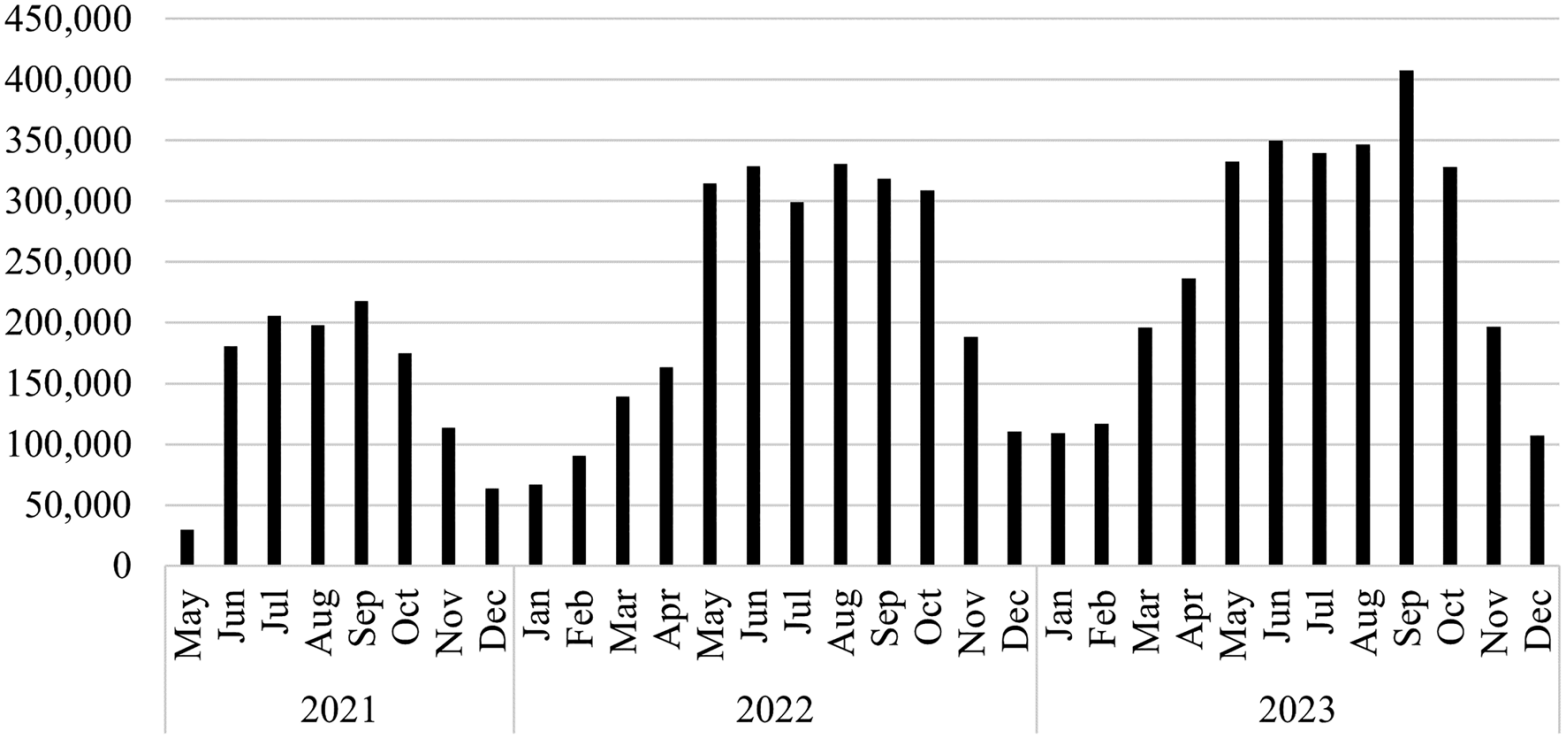
Monthly total number of trips by the Budapest BSS (MOL Bubi).

The Budapest BSS, called MOL Bubi, was opened in September 2014 with 76 stations. The system was gradually increased and reached 160 stations in 2020. However, usage did not increase as expected and the Covid19 pandemic made it clear that a substantial renewal of the system is required^15^. The Bubi 2.0 was launched in May 2021. Since that time, usage increased (Fig. 1), and the system was expanded continuously reaching more than 200 stations and 2000 bikes in 2023. Its operational coverage spans approximately 40 square kilometres. The operation is overseen by BKK Centre for Budapest Transport, the municipal agency responsible for public transport in Budapest, with day-to-day management handled by a third-party operator. For access, users have the option of a monthly pass priced at 500 HUF (equivalent to approximately 1.35 EUR), or an annual pass costing 5000 HUF (13.5 EUR) per year. The prices were increased from January 19, 2023 to 1000 HUF (2.7 EUR) and 8500 HUF (22.9 EUR), respectively. Pass holders enjoy the benefit of the first 30 minutes of each ride being free of charge, with a subsequent fee of 20 HUF (0.05 EUR) per minute applied thereafter. For occasional users, a pay-as-you-go rate is also available, with the same 20 HUF per minute charge. These fees were also increased from January 19, 2023 to 40 HUF (0.11 EUR). Registration and payment for the service can be conveniently made through the accompanying mobile application, compatible with all smartphones.

The seasonality of the BSS usage is substantial in Budapest (Fig. 1). The peak period is between May and October with substantially higher usage in all years. Hence, the off-peak period is between November and April.

**Figure 2:**
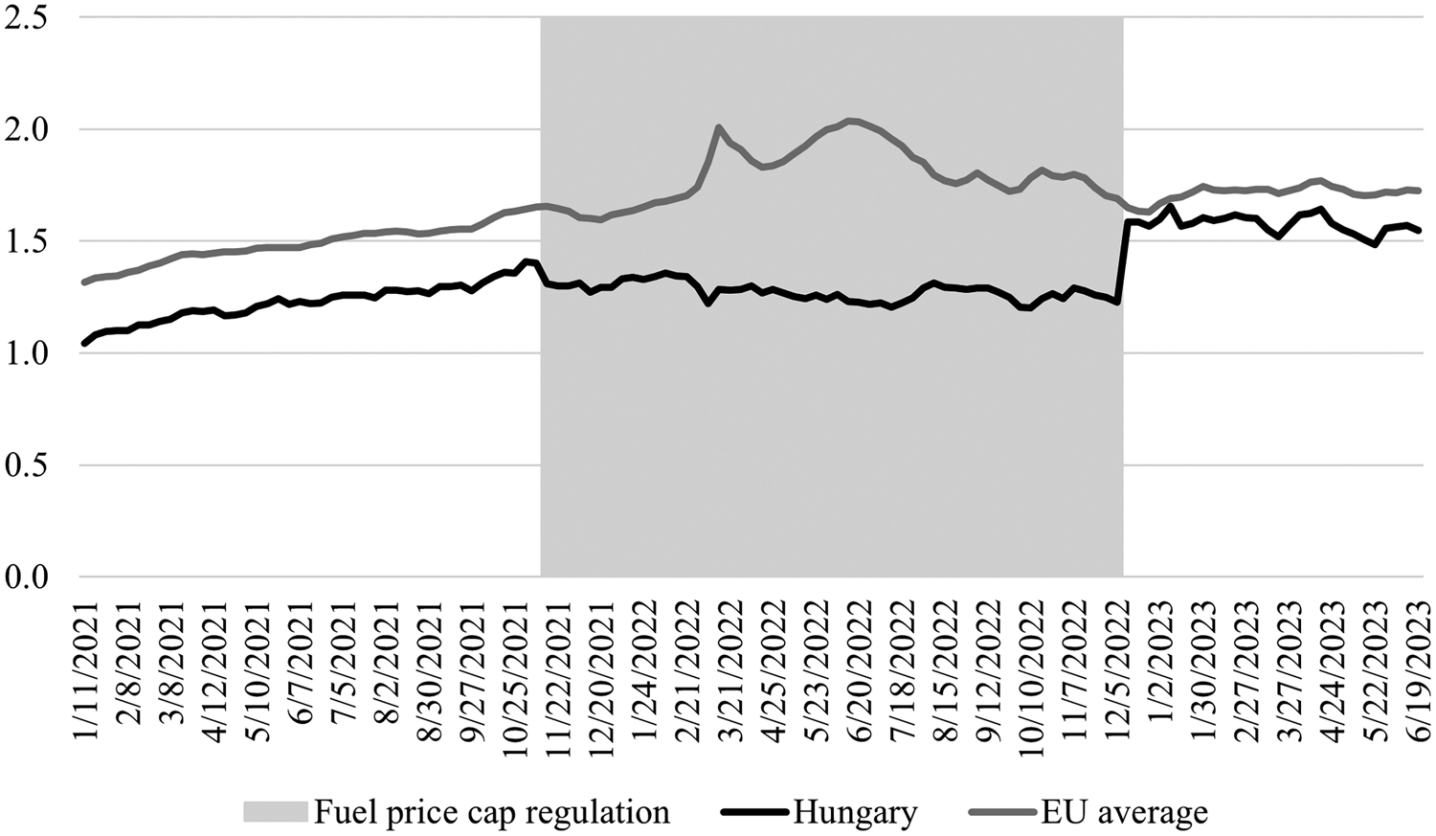
Weekly Euro-super 95 fuel prices with taxes in Hungary and the EU average (€/l). Source: Weekly Oil Bulletin of the European Commission (https://energy.ec.europa.eu/data-and-analysis/weekly-oil-bulletin_en).

Fuel prices are normally not regulated in Hungary, and prices are primarily determined by international crude oil prices, exchange rate movements, and local taxes (mainly value added tax and excise tax). However, as a reaction to increasing inflation and the cost-of-living crisis, the Hungarian government introduced a retail price cap for gasoline and diesel on November 15, 2021, at 480 HUF/litre (1.2–1.4 EUR/litre depending on the exchange rate). In 2021, the price cap was often not effective, or the capped price was only slightly below the market price. However, in 2022, fuel prices increased substantially throughout Europe, leading to a sizeable difference in market and capped prices (Fig. 2).

As a result, importers stopped supplying the Hungarian market, and the only domestic refinery experienced increasingly severe problems in satisfying the increasing demand. Despite several supply disruptions mainly in the countryside, the Hungarian government communicated that the fuel price cap would remain in force at least until the beginning of 2023. This suddenly changed on December 6, 2022, when the price cap was lifted with immediate effect. The sudden elimination of the price cap led to an around one-third increase in fuel prices that remained for a longer time horizon (Fig. 2). After lifting the price cap, fuel prices became similar to EU average prices. In this article, we exploit this sudden and unexpected decision to lift the fuel price cap on BSS usage.

## Data

We combined administrative and survey data to estimate the effect of the substantial fuel price increase on BSS usage. Administrative data contain BSS trips obtained from the system operator company, BKK Centre for Budapest Transport. The dataset contains the start and end time and location of every journey made by the BSS in 2021, 2022 and 2023. The dataset was first cleared by eliminating invalid entries (journeys without starting location, journeys made by the system operator to balance bicycles across stations) and journeys shorter than one minute and longer than three hours. The Budapest BSS is predominantly a docking station-based system; however, it is possible to leave the bikes anywhere within the operation area of the system for a penalty fee of 5000 HUF (13.5 EUR). Due to the rather substantial penalty fee, more than 95% of the trips start and terminate at a docking station. Trips not starting at a docking station were also eliminated from the dataset. The resulting dataset contains 6,907,760 journeys.

The dataset was amended with weather data (average daily temperature, daily total precipitation and daily average wind speed) collected from the NASA Power Data Access Database since weather conditions substantially impact BSS usage^15,22–28^. Based on prior literature, the effect of temperature on BSS usage is not linear, hence, 5 °C intervals were applied.

BSS trip generation is also substantially impacted by the natural and built environment^12,29–34^. Since this rarely changes within a year, we control for the built environment using station-specific dummy variables. Finally, the pricing of the BSS is another important factor of usage^35–37^. The prices were flat in 2021 and 2022 but increased substantially on January 19, 2023, which we also take into consideration in our model.

Finally, the Covid19 pandemic led to the implementation of various measures in numerous countries with the objective of halting the spread of the virus. These measures had a significant impact on mobility^38^ and also influenced BSS usage^15^. In order to control for these measures, the Stringency index from the Oxford Covid19 Government Response Tracker database^39^ was employed. The descriptive statistics of the administrative dataset can be found in Table 1.

**Table 1:** Descriptive statistics of the administrative dataset.

Variable	Obs	Mean	Median	St. dev	Min	Max
Number of BSS trips generated per station on workdays	117,642	45.1	35	36.8	0	585
Number of BSS trips generated per station on weekends	47,278	33.9	23	35.8	0	747
Daily average temperature (°C)	956	12.8	13.0	9.4	− 8.7	31.5
Daily total precipitation (mm)	956	1.8	0.1	4.5	0	78.4
Daily average wind speed (m/s)	956	2.4	2.1	1.1	0.6	8.2
Covid19 Stringency index	956	17.6	11.1	10.7	11.1	51.7

The survey data originate from an online survey jointly executed by the BKK Centre for Budapest Transport and Corvinus University of Budapest between March 24 and April 17, 2023. The survey link was sent via email to the BSS users and shared on the Facebook page of the BSS and the BKK Centre for Budapest Transport. A total of 599 people completed the survey. The respondents are mainly but not exclusively BSS users. The questionnaire contained two questions related to the elimination of the fuel price cap and how respondents altered their commuting habits as a response (if any). Additionally, the survey asked several questions about commuting habits and BSS usage as well as basic demographic characteristics. The descriptive statistics of the relevant variables of the survey can be found in Table 2.

**Table 2:** Descriptive statistics of the relevant survey questions.

Variable	Categories or measurement	Obs	Average or percent (%)	St. dev
Gender	Male	468	78	–
	Female	112	19	–
	n/a	19	3	–
Age	In years	599	35	11.2
Resident	Budapest	514	86	–
	*Out of*: districts with BSS station	412	69	–
	districts without BSS station	102	17	–
	In the agglomeration of Budapest	64	11	–
	Outside	21	4	–
Level of education	Primary school	12	2	–
	Vocational training	7	1	–
	High school graduation	104	17	–
	Technical training	18	3	–
	BA/BSc diploma	184	31	–
	MA/MSc diploma	220	37	–
	Postgrad diploma	30	5	–
	n/a	24	4	–
Employment	Employed	516	86	–
	Student	46	8	–
	Pensioner	4	1	–
	Other	33	6	–
Car ownership	Owns a car and/or has access to a corporate car	338	56	–
	No car access	261	44	–
BSS usage	Daily	92	15	–
	Weekly	341	57	–
	Occasionally	129	22	–
	Not using	37	6	–

## Methodology

The research aims to identify the impact of a very substantial one-time fuel price increase on BSS usage. There are different ways to estimate this effect. Some researchers (e.g., Ref.^18^) included fuel price as an explanatory variable in the regression. However, before lifting the fuel price cap in December 2022, prices did not change, as the capped price was set at all gasoline stations every day. After lifting the cap, fuel prices varied in time, but this variation was rather small compared to the one-time price increase (Fig. 2). Additionally, frequent but smaller fuel price changes can have a less important effect on BSS usage than a very substantial one-time price increase. Therefore, we do not add fuel price as an explanatory variable, but we apply a difference-in-difference (DID) approach similar to, for example, Xabier^40^.

DID is a statistical technique used in the social sciences to estimate the causal effect of a treatment on an outcome^41,42^. DID does this by comparing the difference in the outcome between the treatment group and the control group before and after the treatment is implemented. Hence, the DID method requires two groups of observations, one that is treated and one that is not treated. Since the elimination of the fuel price cap impacted the entire country, we applied a time-lagged control group, namely, the BSS data from the same period of the preceding year. At this time, fuel prices were roughly equal to the fuel prices during the capped period (Fig. 2), hence, this period can serve as an ideal benchmark group. Weather differences might exist across the two years, but we control for these factors.

One crucial assumption of the DID method is the parallel trend assumption, which states that the treatment and the control groups would have followed the same trend in the outcome if the treatment had not been implemented^43^. In this case, the previous year’s usage volume serves as a control group. BSS usage is mainly determined by weather, built and natural environment and prices that we all take into account in our estimation, hence, it is reasonable to assume that the parallel trend assumption holds.

The estimated equation is as follows:

1 $$\begin{aligned}Trip{s}_{it}=&\,{\beta }_{1}HighFuelPricePerio{d}_{t}+{\beta }_{2}TreatedPerio{d}_{t}\\&+{\beta }_{3}HighFuelPricePerio{d}_{t}\times TreatedPerio{d}_{t}+\Gamma {X}_{t}+{c}_{i}\\&+Wee{k}_{t}+Mont{h}_{t}+{u}_{it},\end{aligned}$$

where $Trip{s}_{it}$ refers to the number of trips generated by station $i$ on day $t$, $HighFuelPricePerio{d}_{t}$ refers to the period of the year when the fuel price cap was not in effect (i.e., after December 7 in both 2021 and 2022) and $TreatedPerio{d}_{t}$ takes 1 for the year when the fuel price cap was eliminated (i.e., July 2022–June 2023 period) and 0 for the previous year (i.e., the control group, July 2021–June 2022 period). ${X}_{t}$ contains the weather- and Covid19-related control variables and the price increase dummy variable, while ${c}_{i}$ is the station fixed effect capturing all time invariant variables of station $i$, $Wee{k}_{t}$ and $Mont{h}_{t}$ are week and month fixed effects, respectively, and ${u}_{it}$ is the idiosyncratic error term. ${\beta }_{1}$ shows the average usage difference between before December 6 and after December 7, i.e., the average difference that is observable in these two time periods due to seasonality in every year. ${\beta }_{2}$ indicates the usage difference between the treated period and the control period; hence, this shows the overall usage change from one year to another. The variable of interest is ${\beta }_{3}$, which captures the effect of the fuel price cap elimination on BSS usage.

To analyse the spatial heterogeneity of the fuel price increase, we interacted the difference-in-difference model with the station dummy variables (${c}_{i}$). The approach is similar to a complete structural break except that the Covid19-and the weather-related variables were not interacted with the station dummies, i.e., a homogenous effect was assumed here. The results of this regression can shed light on the differences across the stations.

We estimate trip generation; hence, the dependent variable can only take positive integer values. This requires count data models. According to Jaber and Csonka^44^, the negative binomial model performs better for BSS data than the Poisson model. Other researchers (e.g., Refs.^26,45,46^) also applied negative binomial models to investigate BSS data, as overdispersion is present in the data. However, to take the panel setting into consideration, a fixed effect negative binomial model is the preferred choice. Allison and Waterman^47^ proposed that adding the station dummy variables to the equation can be a good way to account for time-invariant effects that are also supported by simulation results (instead of using the conditional fixed effect negative binomial model estimator). We also follow this approach and estimate Eq. (1) using dummy variable negative binomial regression.

Finally, we estimate different regressions for workdays and weekends, as workday usage is normally connected to commuting, while weekend usage is more related to leisure and sport activities^15,22,25^.

## Results

The analysis is divided into three parts. First, we look at the panel regression results; second, we investigate the spatial distribution of the effect of the fuel price increase on the number of BSS trips; third, we review the survey findings.

### Regression results

The estimation of Eq. (1) was conducted separately for workdays and weekends. Additionally, three distinct time horizons were considered with the objective of investigating the temporal evolution of the effect. Firstly, a brief period of time is considered, approximately one month prior and after the elimination of the fuel price cap (columns (1) and (4) of Table 3). This is the immediate effect, which demonstrates how travellers responded to the fuel price increase immediately following its occurrence. Secondly, the entire off-peak season (November to April) is considered (columns (2) and (5) of Table 3). This is a considerably longer time horizon, but since the off-peak and peak seasons may exhibit disparate characteristics, it may be beneficial to consider them separately. This is referred to as the first short-term effect, as it encompasses a limited period of time following the fuel price increase. Thirdly, a full year is investigated (columns (3) and (6) of Table 3) in order to estimate a somewhat longer-term, but still short-term, effect.

**Table 3:** Negative binomial regression results.

Independent variables	(1)	(2)	(3)	(4)	(5)	(6)
	Workday			Weekend		
	Immediate (Nov–Dec)	Short-term		Immediate (Nov–Dec)	Short-term	
		5 months (Nov–Apr)	7 months (Jul–Jun)		5 months (Nov–Apr)	7 months (Jul–Jun)
High fuel price period × Year	0.058*** (0.013)	0.023** (0.010)	0.021*** (0.008)	0.252*** (0.039)	− 0.086*** (0.022)	0.092*** (0.017)
High fuel price period	− 0.148*** (0.018)	− 0.120*** (0.017)	− 0.124*** (0.017)	− 0.787*** (0.054)	− 0.007 (0.028)	− 0.147*** (0.026)
Year	− 0.086 (0.076)	0.307*** (0.012)	0.196*** (0.008)	− 0.365** (0.183)	0.575*** (0.024)	0.256*** (0.017)
Temperature < -5°C	–	− 0.508*** (0.033)	− 0.486*** (0.033)	–	–	–
Temperature between -5°C and 0°C	− 0.167*** (0.009)	− 0.214*** (0.006)	− 0.207*** (0.006)	− 0.090*** (0.023)	− 0.137*** (0.013)	− 0.145*** (0.012)
Temperature between 5°C and 10°C	0.027** (0.011)	0.087*** (0.005)	0.085*** (0.005)	0.328*** (0.026)	0.335*** (0.013)	0.344*** (0.012)
Temperature between 10°C and 15°C	0.288*** (0.024)	0.314*** (0.007)	0.280*** (0.007)	–	0.572*** (0.020)	0.547*** (0.016)
Temperature between 15°C and 20°C	–	–	0.420*** (0.010)	–	–	0.799*** (0.019)
Temperature between 20°C and 25°C	–	–	0.432*** (0.011)	–	–	0.893*** (0.022)
Temperature > 25°C	–	–	0.407*** (0.011)	–	–	0.841*** (0.024)
Precipitation	− 0.039*** (0.001)	− 0.040*** (0.001)	− 0.036*** (0.000)	− 0.030*** (0.002)	− 0.035*** (0.002)	− 0.035*** (0.001)
Wind speed	− 0.012*** (0.004)	− 0.029*** (0.002)	− 0.023*** (0.001)	0.005 (0.008)	− 0.049*** (0.004)	− 0.037*** (0.003)
Covid19 related stringency	− 0.024 (0.004)***	− 0.004*** (0.000)	− 0.009*** (0.000)	− 0.039*** (0.009)	0.006*** (0.001)	− 0.002** (0.001)
Price increase	–	− 0.106*** (0.010)	− 0.080*** (0.010)	–	− 0.269*** (0.021)	− 0.232*** (0.020)
Constant	4.791*** (0.118)	3.757*** (0.030)	3.983*** (0.026)	4.575*** (0.287)	3.144*** (0.051)	3.561*** (0.038)
ln(alpha)	− 2.990*** (0.024)	− 2.860*** (0.012)	− 2.806*** (0.007)	− 2.528*** (0.037)	− 2.280*** (0.017)	− 2.400*** (0.011)
*N*	14,092	41,680	85,040	5,937	18,744	36,953

All regressions contained station, week, and month fixed effects; the reference category for temperature is between 0 °C and 5 °C. Standard errors in parentheses.

*p < 0.1; **p < 0.05; ***p < 0.01.

The high fuel price period covers the time after the elimination of the price cap, i.e., from December 7 to 31 in the immediate sample (columns (1) and (4) of Table 3), from December 7 to April 30 in first short-term sample (columns (2) and (5) of Table 3) and from December 7 to June 30 in second short-term sample (columns (3) and (6) of Table 3) in both years. The results confirm that BSS usage is *ceteris paribus* lower at that time by 12–15% on workdays. BSS usage in Budapest is highly seasonal and Fig. 1 also confirms that usage is generally lower between December and June.

The overall usage of BSS was higher in the 2022/2023 period in comparison to the previous year. This is also consistent with the findings presented in Fig. 1. The increase was approximately 20–30%, depending on the time horizon considered. Nevertheless, no discernible difference was observed during weekdays in the immediate sample, while a decline was apparent during weekends.

The coefficients for temperature, precipitation, and wind speed are consistent with expectations. Lower temperatures, higher precipitation and higher wind speeds are *ceteris paribus* associated with reduced BSS usage. A daily average temperature exceeding 25 °C is associated with a reduction in BSS usage compared to a daily average temperature between 15 and 20 °C. This finding is consistent with previous research (e.g., Refs.^22,26^). Moreover, the implementation of more stringent measures related to the Covid19 pandemic has led to a reduction in BSS usage. This may be attributed to a decline in the overall number of travel needs.

The price increase that occurred in January 2023 significantly decreased BSS usage, which is also in line with expectations. The reduction was more than twice as high on weekends, which is likely due to the fact that weekend usage is more flexible and price sensitive.

The variable of interest, the additional change in BSS usage after the fuel price cap elimination, yielded significantly positive effects. This variable represents the average change in trip generation after the fuel price cap elimination, taking into account (i) the overall usage difference between the years; and (ii) the seasonality of the BSS based on the previous year’s data. According to the immediate sample, BSS usage increased by 5.8% on workdays. For weekends, the short-term effect was larger, approximately 25.2%. The results show a significant and positive increase in workday BSS usage in the short-term samples. However, the effect size is approximately half of the immediate effect (~ 2%). This indicates that while a portion of the increased BSS demand was transient, approximately half of this increase persisted over a longer period.

Surprisingly, the short-term effect during weekends is negative. However, this is a less reliable sample due to the limited number of weekends included in the price cap period (from November 1 to December 7).

### Spatial distribution

**Figure 3:**
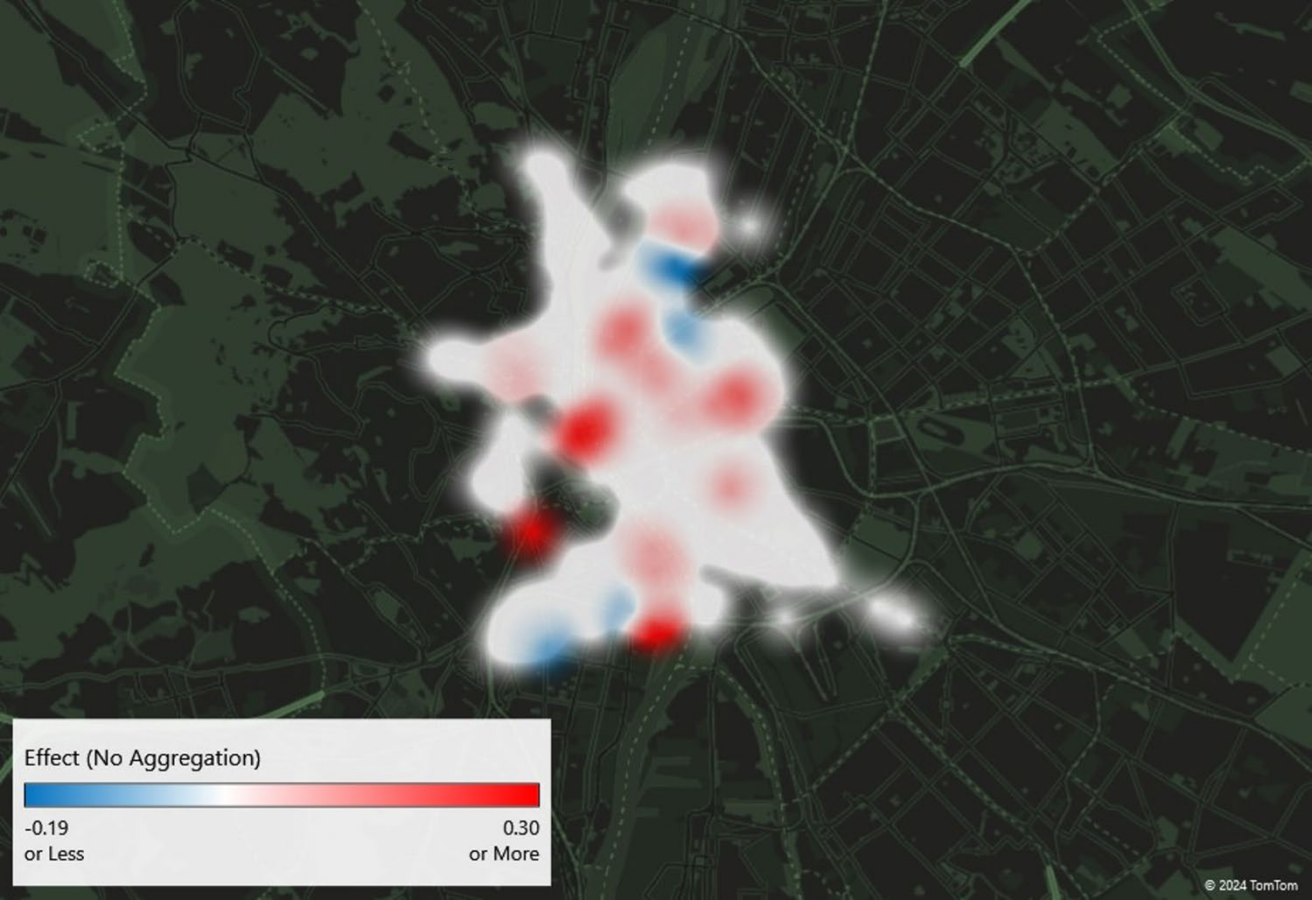
The effect of the fuel price cap elimination on BSS trip generation by stations based on the immediate sample. Docking stations marked with white indicate no significant change, red indicates a significant increase and blue indicates a significant decrease in BSS usage.

The spatial pattern of the change was investigated for the workday subsample only since 76% of the trips were made during workdays. Figure 3 shows the variable of interest (${\beta }_{3}$ in Eq. (1)) by docking station on the map based on the immediate sample. White areas indicate no significant change, while red indicates a significant increase and blue indicates a significant decrease in BSS usage because of the fuel price cap elimination. Several stations did not experience any significant changes. However, some stations mainly located on the boarder of the operating area increased their trip generation. A higher number of trips in the outer areas indicates that commuters most likely changed from car to BSS for the last part of their trip.

However, some stations (also in the outer areas of the city) experienced a decline in trip generation that can be due to changing commuting habits. Combining cars and BSS is a viable option, but the fuel price increase can encourage commuters to change their commuting habits completely and switch, for example, to public transport. This can reduce BSS usage as well.

**Figure 4:**
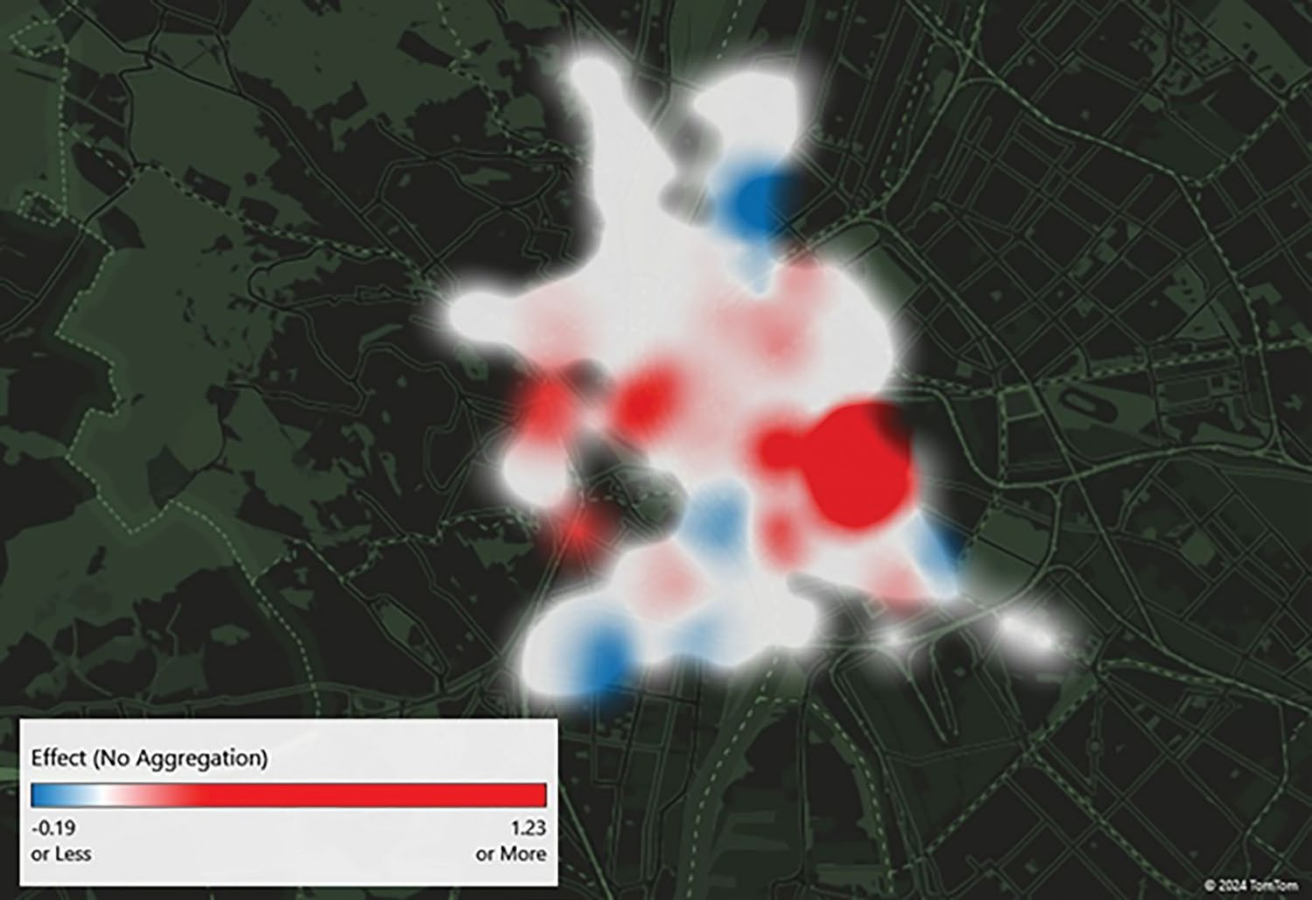
The effect of the fuel price cap elimination on BSS trip generation by stations in the short term (5 months). Docking stations marked with white indicate no significant change, red indicates a significant increase and blue indicates a significant decrease in BSS usage.

Figure 4 visualizes the short-term effects using the sample from November to April. The by-station changes are more evenly distributed across the city, but one can observe a decline in usage in several parts of the city. The increased usage is particularly substantial in the southeast part of the city, but out of the 176 stations, 25 (14%) experienced an increase and 10 (6%) experienced a decrease in usage. The average percentage increase was higher than the average percentage decrease, which is the reason behind the overall positive effect of the fuel price increase on BSS usage presented in the previous subsection.

**Figure 5:**
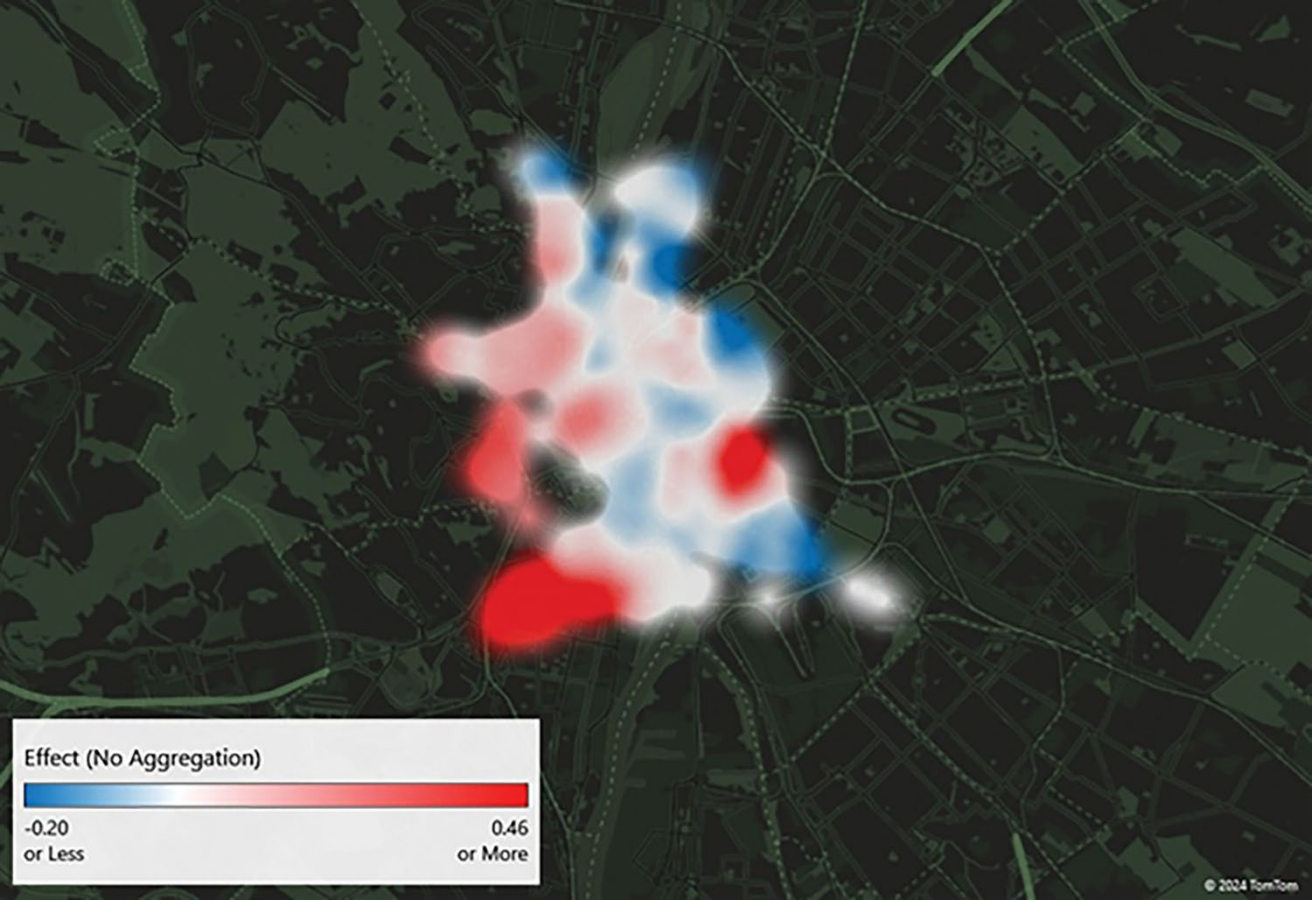
The effect of the fuel price cap elimination on BSS trip generation by stations in the short term (7 months). Docking stations marked with white indicate no significant change, red indicates a significant increase and blue indicates a significant decrease in BSS usage.

With regard to the short-term effects observed 7 months after the elimination of the fuel price cap (Fig. 5), it is evident that commuters exhibited a range of responses at various locations within the city. While some locations exhibited a notable increase in usage, others demonstrated a significant decline. Nevertheless, as was observed in the 5 months sample, the increases are greater than the decreases, resulting in an overall positive impact.

As evidenced by the spatial analysis, the fuel price increase resulted in a number of changes in trip generation. Some stations were favoured by the change, while others experienced a decline in volume. This can be attributed to the fact that a number of individuals combine cars and BSS in Budapest^48^, and may therefore modify their daily commuting habits in response to higher fuel prices. A reduction in the use of private vehicles by this subpopulation may also result in a decline in their BSS usage.

### Survey results

The regression results are also supported by the findings of the survey. Of the respondents, 56% indicated that they own a car, and 25% of them reduced their car usage in the city following the fuel price increase. The majority (91%) maintained this at least until the time of the survey (i.e., March/April), and approximately half of them (51%) increased their BSS usage as a mitigation strategy. This indicates that 6.5% of the respondents increased their BSS usage following the substantial fuel price increase. While 69% of the respondents reside in a district with a BSS station, 63% of those who increased their BSS usage live in one of these districts. Since 37% does not have a BSS station close to its residence, they combined BSS more frequently than they did previously.

Those who reduced their car usage as a response to the fuel price cap elimination indicated that they use the BSS at least on a weekly basis (only 1 respondent indicated that he uses the BSS only occasionally), mainly to commute to work and to go shopping or for administration. Two-thirds of them still use their car on a weekly basis in the city; therefore, they were not completely discouraged from using a car and only reduced their usage.

Conversely, 10% of the respondents who reduced their car usage combined BSS and cars in a single trip. Apart from 1 respondent, all of them reside either outside of Budapest or in a district without a BSS station. Consequently, a reduction in car usage may result in a decrease in BSS usage as well, which can lead to the BSS usage reductions observed in Figs. 3, 4 and 5.

The survey and the empirical findings indicate that a small proportion of commuters switched from car to BSS, and that some of them were able to maintain this change for a longer period of time. As time progressed, the positive change may have been less sustained, with the immediate effect being larger than the short-term one. This is also consistent with the regression results.

## Discussion

This paper examined the impact of a significant increase in fuel prices on BSS usage. Using a difference-in-difference approach, the results show a 2% to 6% increase in usage on workdays, which is also reinforced by survey findings. Previous research^13,18–20,49^ has also demonstrated that higher fuel prices are positively affecting BSS usage. However, the increase is often low.

The findings of this study are notable for their uniqueness. Despite the substantial one-time price increase, the increase in BSS usage (on workdays) was relatively modest, amounting to 2% seven months after the removal of the fuel price cap. In contrast, the fuel price increase was considerable, reaching approximately 33%. This is considerably larger than the typical increase observed in developed countries. These results indicate that fuel price increases can only have a marginal impact on BSS usage. In the case of Budapest, seasonality, BSS ticket and pass prices, and the overall upwards trend of the BSS have much more substantial effects on BSS usage. As demonstrated by Berezvai^15^, positive user experience can be a much larger driver of BSS usage than the effect identified in this research following the fuel price increase. Furthermore, BSS usage is less prevalent than car usage. Consequently, the 2% increase in BSS usage does not result in a comparable reduction in car usage. Consequently, the impact on reducing the number of cars is minimal.

On the other hand, this research investigated only the change from car to BSS. Higher fuel prices might encourage commuters to use their own bikes or public transport or scooter^20^. Hence, a fuel price increase might have other sustainability-related benefits that future research can investigate and quantify.

The encouragement of commuters to rely more on the BSS for their journeys within the city can result in a number of individual and social benefits, while also contributing to the achievement of the 11th SDG. The expansion of BSS usage can yield a number of benefits for sustainable urban communities^50^, since it makes cycling available for everyone, as there is no need to own (and store) a bicycle, it makes it possible to easily combine different transportation modes within a single journey and within a whole day; as well as it can be easily used by tourists. In terms of the benefits of cycling, firstly, it can assist in the reduction of traffic congestion by reducing the number of vehicles on the road. Secondly, the use of bicycles can contribute to the creation of a more liveable and sustainable urban environment^51,52^ with improved air quality. Thirdly, it can facilitate physical activity, which will have a beneficial impact on public health.

Policymakers can achieve these benefits in several ways. For instance, they can build more bicycle lanes^12^ or improve the user experience of the BSS^15^. Our findings indicate that indirect measures, such as higher gasoline prices, can also lead to higher BSS usage. However, the impact of this is rather minor, suggesting that policymakers should consider other options with a larger potential effect.

## Conclusions, limitations, and future research

The results of this study suggest that there is a positive, albeit minor, relationship between fuel prices and BSS usage. This is consistent with previous research, which has found that higher fuel prices lead to an increase in the demand for alternative transportation modes, such as bicycle sharing^49^. The increase in BSS ridership after the elimination of the fuel price cap was significant but rather minor in absolute terms. This suggests that fuel prices are not the only (and most likely, not the most important) factor that influences BSS ridership. Other factors, such as the configuration of the BSS^53^, bicycle-related infrastructure and network^54^, weather, and the overall level of urbanization, play more important roles.

The geographical pattern of the change shows that BSS usage increased in several parts of the city, mainly in the outer districts; however, some docking stations experienced a decline. The survey findings also support the conclusion that the elimination of the fuel price cap led to an overall increase in BSS ridership, but some people might have reduced their BSS usage because they had previously combined car and BSS.

However, it is important to note that local factors also play a role in BSS utilization and that the effect of fuel prices may vary depending on the specific context. This study was conducted in a single city, Budapest, Hungary. Budapest is a large, relatively affluent city with a well-established BSS and continuously expanding bicycle infrastructure. It is possible that the results would be different in smaller, less affluent cities with less developed BSS and bicycle infrastructures.

Finally, it is important to note that the treatment effect may change over time. This analysis considered three periods; the immediate period showed a larger impact of the fuel price increase on BSS usage than the longer periods. Taking even longer periods into consideration, the effect might further diminish. Despite these limitations, the study provides valuable insights into the relationship between fuel prices and BSS usage. Future research should examine the effect of fuel prices on BSS ridership in other cities and over a substantially longer period of time.

## Data Availability

The datasets generated and analysed during the current study are available from the corresponding author on reasonable request.
